# Comparative Evaluation of Local Hemostatic Agents in Minor Oral Surgical Procedures: A Randomized Clinical Trial

**DOI:** 10.7759/cureus.85754

**Published:** 2025-06-11

**Authors:** Kshitija Patil, Jay N Goyal, Saurabh Dudhe, Janice John, Simona Joseph, Sanchi Kadbe, Seema Gupta

**Affiliations:** 1 Department of Oral and Maxillofacial Surgery, Jawahar Medical Foundation’s Annasaheb Chudaman Patil Memorial Dental College, Dhule, IND; 2 Department of Oral and Maxillofacial Surgery, Swargiya Dadasaheb Kalmegh Smruti Dental College and Hospital, Nagpur, IND; 3 Department of Orthodontics, Kothiwal Dental College and Research Centre, Moradabad, IND

**Keywords:** extraction, hemostasis, oral surgical procedures, surgical, tooth

## Abstract

Background

Achieving effective hemostasis is a critical aspect of minor oral surgeries. This study aimed to compare the efficacy of Botroclot, chitosan, adrenaline, and tranexamic acid in achieving hemostasis and preventing postoperative bleeding, thereby identifying the most clinically efficient hemostatic method for routine use in oral surgery.

Methodology

A parallel-group randomized controlled clinical trial was conducted at the Department of Oral and Maxillofacial Surgery between February and April 2025. A total of 60 healthy patients aged 20-50 years who required extraction of the upper or lower first or second molars were randomly allocated into five groups (n = 12). Group 1 was treated with Botroclot. Group 2 was treated with chitosan dressing. Group 3 was treated with adrenaline. Group 4 was treated with tranexamic acid. Group 5 (control) was treated with pressure gauze soaked in normal saline. All procedures were performed under local anesthesia. The hemostatic agent was applied to the socket post-extraction, and the time to achieve hemostasis (observation time) was recorded. Preoperative bleeding time, clotting time, and the incidence of postoperative rebleeding were also evaluated. Statistical analysis was conducted using one-way analysis of variance (ANOVA), Tukey’s post-hoc test, and Pearson’s correlation. Statistical significance was set at p-values <0.05.

Results

All groups were comparable at baseline. The shortest mean observation time was recorded in the Botroclot group, followed by that in the tranexamic acid and chitosan groups. The longest was in the control group (1.80 ± 0.41 minutes). ANOVA revealed a statistically significant difference in observation time among the groups (p = 0.001). Post-hoc analysis revealed significant differences, particularly between the Botroclot, adrenaline, and pressure gauze groups. No postoperative complications or bleeding events were noted. Significant correlations between bleeding and observation time were observed in the adrenaline, tranexamic acid, and control groups.

Conclusions

Botroclot demonstrated the most rapid hemostatic effect, followed by tranexamic acid and chitosan. Pressure gauze and adrenaline were less effective. These findings support the use of Botroclot and tranexamic acid as efficient hemostatic agents in minor oral surgery.

## Introduction

Hemostasis plays a vital role in the success of minor oral surgical procedures by ensuring a dry and visible operative field, reducing intraoperative complications, and promoting optimal wound healing [1]. Common procedures such as dental extractions, biopsies, and alveoloplasty frequently involve soft tissue manipulation and minor bleeding [2]. Although bleeding in healthy individuals is generally self-limiting, certain clinical situations, such as bleeding tendencies, anticoagulant therapy, or surgeries involving highly vascular tissues, require the use of additional hemostatic measures [2]. Traditional techniques such as pressure application and suturing, which are effective in many cases, may not always offer rapid or sufficient bleeding control [1]. Moreover, surgical patients exhibiting undiagnosed elevations in the international normalized ratio (INR) are predisposed to increased instances of postoperative hemorrhage. Unregulated hemorrhage and the ensuing state of shock are the principal factors contributing to fatalities associated with combat [3,4]. This has led to the development and increasing use of various local hemostatic agents designed to provide efficient and targeted hemostasis at the surgical site.

Local hemostatic agents are substances applied directly to bleeding tissues to control hemorrhage by promoting clot formation, enhancing platelet aggregation, inducing vasoconstriction, or stabilizing the formed clot [2]. Several agents have been introduced into clinical practice, each of which functions via distinct mechanisms. Botroclot is a commercial topical formulation comprising a sterile hemocoagulase solution at a concentration of 0.2 colony units (CUs), derived from the venom of *Bothrops atrox* or *Bothrops jararaca*, along with chlorhexidine at a concentration of 0.1% v/v. Hemocoagulase is an enzyme typically present in the venom of hemotoxic serpents that facilitates blood coagulation. Its enzymatic function is linked to a specific molecule known as Batroxobin (BTX) [5]. BTX has been demonstrated to enhance wound healing in dental surgical procedures by facilitating collagen deposition while simultaneously minimizing inflammation and the risk of infection [6].

Chitosan is a naturally derived polysaccharide obtained from crustacean shells. It exhibits unique mucoadhesive and hemostatic properties. Upon application, chitosan interacts with red blood cells and platelets to form a gel-like clot independent of the intrinsic coagulation pathways of the body. This makes it particularly useful in patients with clotting disorders or those receiving anticoagulant therapy [7]. Adrenaline functions as a vasoconstrictive agent that can be topically applied to achieve hemostasis. This compound is classified as a catecholamine, which interacts with both alpha- and beta-adrenergic receptors. The activation of alpha-1 receptors induces various effects on the sympathetic nervous system. Primarily associated with hemostasis, stimulation of this receptor enhances the contraction of arteriolar smooth muscle, thereby decreasing the rate of blood flow [8]. The hemostatic properties of 1:1,000 adrenaline warrant consideration as a viable alternative in operative dentistry and oral surgical procedures to facilitate hemostasis and enhance intraoperative visibility, which subsequently diminishes the likelihood of iatrogenic injury and hemorrhage, thus optimizing treatment results [9].

Tranexamic acid is a synthetic antifibrinolytic agent that helps stabilize blood clots by inhibiting plasminogen activation [10]. It prevents the breakdown of fibrin, the main protein component of blood clots, thus promoting clot stability and prolonging hemostasis. Its topical application has proven to be effective, especially in controlling postoperative bleeding [11]. Although tooth extraction is regarded as a secure procedure for individuals with an elevated risk of hemorrhage, instances of postoperative bleeding have been documented [12]. Consequently, it is imperative to identify the most effective adjunctive strategy to achieve enhanced hemostatic control. Therefore, this study aimed to compare the efficacy of various local hemostatic agents, namely, Botroclot, chitosan, adrenaline, and tranexamic acid, with that of conventional pressure application using gauze soaked in normal saline to achieve hemostasis during minor oral surgical procedures. The primary objective of this study was to compare the efficacy of various local hemostatic agents in achieving hemostasis by measuring the observation time required to stop bleeding after tooth extractions in minor oral surgery. The secondary objectives were to assess the incidence of postoperative rebleeding, evaluate clot stability through direct observation and patient-reported outcomes when applicable, and confirm baseline hemostatic function by examining preoperative bleeding and clotting times across the five groups.

## Materials and methods

Study design and setting

This study was a parallel-group randomized controlled clinical trial (RCT) conducted in the Department of Oral and Maxillofacial Surgery, Jawahar Medical Foundation’s Annasaheb Chudaman Patil Memorial Dental College, Dhule, from February 2025 to April 2025. Ethical approval (EC/NEW/INST/2022/2959/140), dated 18-12-2024, was obtained from the Institutional Ethics Committee before the initiation of the study. The study was registered with the Clinical Trials Registry of India (CTRI/2025/02/087964), ensuring compliance with the ethical and regulatory standards. All patients were informed of the nature of the study, and written informed consent was obtained from all patients. This study was conducted in accordance with the principles of the Declaration of Helsinki.

Sample size estimation

The required sample size was calculated using the G*Power software (version 3.1.9.2, Heinrich-Heine University, Düsseldorf, Germany). A one-way analysis of variance (ANOVA) test (fixed effects, omnibus) was used to compare bleeding times among the five groups. Based on an effect size of 1.08 from previous studies [13,14], with an α of 0.05, and power (1-β) of 95%, the minimum total sample size required was 60 (12 patients per group).

Patient eligibility

A total of 60 systemically healthy men and women aged 20-50 years were included in this study. Patients who required extraction of the upper or lower first or second molars for periodontal, carious, or traumatic reasons were selected. Preoperative bleeding time (BT), clotting time (CT), INR, and complete blood count (CBC) were measured, and only patients with values within the normal range were included. Patients on anticoagulant or antiplatelet therapy, with bleeding disorders or systemic illnesses, and those allergic to Botroclot, chitosan, adrenaline, and tranexamic acid were excluded.

Randomization, allocation concealment, and blinding

Randomization was performed using a computer-generated sequence created with a random number generator in R software (version 4.3.2, R Foundation for Statistical Computing, Vienna, Austria) to ensure an unpredictable 1:1:1:1:1 allocation of patients to one of five groups (Botroclot, chitosan, adrenaline, tranexamic acid, or control). Allocation concealment was implemented using sequentially numbered, opaque, sealed envelopes (SNOSE), prepared by an independent statistician and stored in a locked cabinet accessible only to a designated trial coordinator, preventing tampering and ensuring investigators remained unaware of group assignments until allocation. Blinding of the operator was not feasible due to the distinct application methods of the interventions (such as topical agents vs. gauze); however, outcome assessors and data analysts were blinded to group assignments to minimize detection and observer bias. Patients were not informed of their allocated intervention to reduce expectation bias, with standardized post-extraction instructions provided to mitigate potential unblinding risks from visible differences in treatment application.

Group allocation

The study participants were randomly divided into five groups, each comprising 12 patients. Group 1 received a Botroclot (Hemocoagulase; Biological E. Limited, Hyderabad, India). Group 2 was treated with a chitosan dressing (Celox™, Medtrade Products Ltd., Crewe, United Kingdom). Group 3 received adrenaline (1:1,000 epinephrine; Neon Laboratories Ltd., Mumbai, India). Group 4 was administered tranexamic acid (Tranexa; Macleods Pharmaceuticals Ltd., Mumbai, India). Group 5 served as the control group and was managed with a pressure gauze dipped in normal saline (0.9% sodium chloride injection IP; Baxter India Pvt. Ltd., Gurgaon, India).

Technique

Preoperative BT and CT were assessed using standard laboratory tests. BT was measured by the Ivy method, observing the time taken for bleeding to stop after a standardized skin puncture. CT was evaluated using the capillary tube method, recording the time for blood to clot in a capillary tube. Both tests ensured patients had normal hemostatic function before inclusion. All procedures were performed under aseptic conditions. Local anesthesia was administered using 2% lidocaine hydrochloride with 1:80,000 adrenaline (Lignox™, Indoco Remedies Ltd., Mumbai, India). The tooth extraction was performed as atraumatically as possible. Following extraction, the patients received the respective hemostatic agents based on their allocated groups. The agent was applied to the extraction socket by using sterile gauze without external pressure. The time required to achieve hemostasis was recorded by direct observation at intervals of 15 seconds until complete cessation of bleeding was noted. The socket was re-evaluated after 30 minutes for any signs of rebleeding.

The primary outcome was the time required to achieve hemostasis (observation time). The secondary outcomes included postoperative rebleeding and clot stability (assessed through observation and patient-reported outcomes, if applicable). Preoperative CT and BT were also considered to validate baseline hemostatic function. To ensure consistency and reliability, all the clinical procedures were performed by a single experienced oral surgeon. The outcome assessment was conducted by an independent observer who was blinded to the group allocation.

Statistical analysis

The collected data were analyzed using SPSS software (version 26.0, IBM Corp., Armonk, NY, USA). Descriptive statistics were used to summarize demographic details and baseline characteristics. The normality of the data was checked using the Shapiro-Wilk test, and the data were found to be normally distributed. One-way ANOVA was used to compare the mean BT, CT, and observation times across the five groups. The post-hoc Tukey’s test was performed for intergroup comparisons. Pearson’s correlation test was used to assess the correlation between the preoperative bleeding time and postoperative observation time. Statistical significance was set at p-values <0.05.

Harms and interim analysis

No major adverse events were anticipated or observed. Unforeseen complications were documented and appropriately managed. An interim analysis was planned to monitor safety and efficacy trends, but was not required as the study progressed without adverse outcomes.

## Results

**Figure 1:**
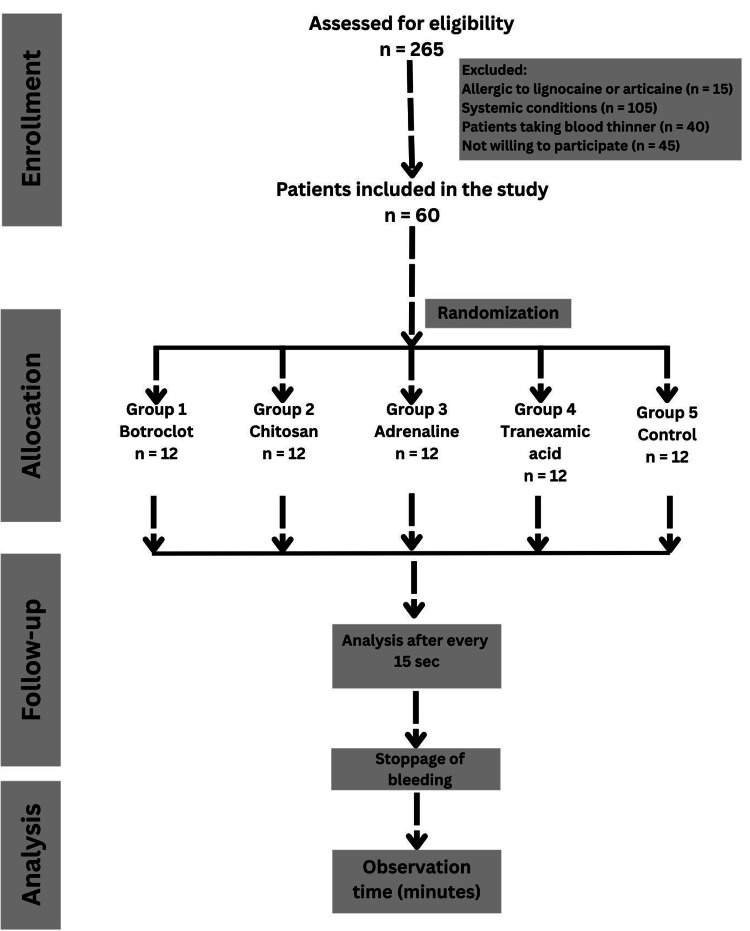
Consolidated Standards of Reporting Trials (CONSORT) flow diagram.

The flow diagram of the Consolidated Standards of Reporting Trials (CONSORT) flow diagram is shown in Figure 1.

Overall, the sex distribution was fairly balanced across the study groups, with each group comprising both male and female participants. The percentages indicated that there was a slightly higher proportion of males in the chitosan and adrenaline groups than in the other groups (Table 1).

**Table 1:** Sex distribution of patients in study groups. Data is presented in the form of frequency and percentage, n (%).

Groups		Botroclot	Chitosan	Adrenaline	Tranexamic acid	Pressure gauze
Sex	Female	6 (10.0%)	4 (6.7%)	4 (6.7%)	5 (8.3%)	5 (8.3%)
	Male	6 (10.0%)	8 (13.3%)	8 (13.3%)	7 (11.7%)	7 (11.7%)

The results indicated that while the overall age distribution among the study groups was similar, there were slight differences in the variability of ages within each group, as evidenced by the differing standard deviations (Table 2).

**Table 2:** Mean age (years) of patients in the study groups. Data is presented in the form of mean ± standard deviation (SD). N: number of patients; CI: confidence interval.

Groups	N	Minimum	Maximum	95% CI for mean	Mean ± SD
Botroclot	12	26	49	32.51–41.49	37.00 ± 7.07
Chitosan	12	20	50	30.62–43.55	37.08 ± 10.18
Adrenaline	12	25	48	31.38–39.78	35.58 ± 6.61
Tranexamic acid	12	21	49	29.37–41.30	35.33 ± 9.38
Pressure gauze	12	26	49	32.36–41.80	37.08 ± 7.43

In terms of BT, the minimum mean value was 2.27 ± 0.17 minutes observed in the chitosan group, and the maximum mean value was 2.48 ± 0.27 minutes found in the adrenaline group. Regarding CT, the minimum mean value was 4.85 ± 0.66 minutes in the adrenaline group, while the maximum mean value was 5.17 ± 0.73 minutes in the Botroclot group. The ANOVA test resulted in non-significant p-values for BT and CT at baseline for all groups, suggesting that all groups were comparable at baseline with no potential bias. For observation time, after the application of the hemostatic agents, the minimum mean value was observed in the Botroclot group, followed by the tranexamic acid, chitosan, and adrenaline groups. The maximum mean value was 1.80 ± 0.41 minutes in the pressure gauze group. The F-value was 15.45 with a highly significant p-value of 0.001, indicating a significant difference in the observation time between the groups. No postoperative rebleeding or complications were noted in any of the groups (Table 3).

**Table 3:** Comparison of various parameters between groups by one-way analysis of variance (ANOVA) test. Data is presented in the form of mean ± standard deviation (SD); *: p-value < 0.05: significant. CI: confidence interval

Groups	Bleeding time at baseline (minutes)		F-value	P-value	Clotting time at baseline (minutes)		F-value	P-value	Observation time after application of hemostatic agents (minutes)		F-value	P-value
	95% CI for mean	Mean ± SD			95% CI for mean	Mean ± SD			95% CI for mean	Mean ± SD		
Botroclot	2.24–2.41	2.33 ± 0.14	2.32	0.681	4.70–5.64	5.17 ± 0.73	0.41	0.803	0.65–1.08	0.87 ± 0.33	15.45	0.001*
Chitosan	2.16–2.38	2.27 ± 0.17			4.32–5.41	4.87 ± 0.86			0.96–1.30	1.13 ± 0.27		
Adrenaline	2.30–2.65	2.48 ± 0.27			4.43–5.27	4.85 ± 0.66			1.23–1.54	1.38 ± 0.25		
Tranexamic acid	2.23–2.43	2.33 ± 0.15			4.65–5.43	5.04 ± 0.62			0.68–1.18	0.93 ± 0.39		
Pressure gauze	2.20–2.39	2.29 ± 0.15			4.57–5.40	4.99 ± 0.66			1.54–2.06	1.80 ± 0.41		

Post-hoc analysis revealed significant differences in the observation time between Groups 1 and 3 (p = 0.004), Groups 1 and 5 (p = 0.001), Groups 2 and 5 (p = 0.001), Groups 3 and 4 (p = 0.014), and Groups 3 and 5 (p = 0.027). Group 4 also showed significant differences from Group 5 (p = 0.001). Non-significant comparisons included Group 1 versus Group 2 (p = 0.331), Group 4 (p = 0.992), Group 2 versus Group 3 (p = 0.371), and Group 4 (p = 0.593). These results highlighted that pressure gauze and adrenaline had the most distinct effects on observation time compared with other hemostatic agents (Table 4).

**Table 4:** Post-hoc analysis of observation time by Tukey’s test. Group 1 received Botroclot, Group 2 was treated with chitosan dressing, Group 3 received adrenaline (1:1,000), Group 4 was administered tranexamic acid, and Group 5 served as the control group and was managed with pressure gauze dipped in normal saline (0.9% sodium chloride); *: p-value < 0.05: significant. CI: confidence interval

Pairwise comparison		t-value	95% CI for mean lower	95% CI for mean upper	P-value
Group 1	Group 2	0.260	-0.1269	0.6469	0.331
Group 1	Group 3	0.510	0.1231	0.8969	0.004*
Group 1	Group 4	0.060	-0.3269	0.4469	0.992
Group 1	Group 5	0.930	0.5431	1.3169	0.001*
Group 2	Group 3	0.250	-0.1369	0.6369	0.371
Group 2	Group 4	-0.200	-0.5869	0.1869	0.593
Group 2	Group 5	0.670	0.2831	1.0569	0.001*
Group 3	Group 4	-0.450	-0.8369	-0.0631	0.014*
Group 3	Group 5	0.420	0.0331	0.8069	0.027*
Group 4	Group 5	0.870	0.4831	1.2569	0.001*

Significant correlations between BT and observation time were found in three groups: adrenaline (p = 0.018), tranexamic acid (p=0.025), and pressure gauze (p = 0.035). No significant correlations were observed for Botroclot (p = 0.318) or chitosan (p = 0.872). The p-values indicated that adrenaline and tranexamic acid showed time-dependent hemostatic effects, whereas pressure gauze demonstrated expected passive bleeding dependence. The non-significant p-values for Botroclot and chitosan suggested that their mechanisms might be less influenced by time (Table 5).

**Table 5:** Pearson correlation of bleeding time and observation time in study groups. *: p-value < 0.05: significant, negative r value indicates negative correlation, very weak correlation: 0.0<∣r∣<0.20; weak: 0.2≤∣r∣<0.4; moderate: 0.4≤∣r∣<0.6; strong: 0.6≤∣r∣<0.8.

Groups	Correlation (r value)	P-value
Botroclot	0.21	0.318
Chitosan	0.05	0.872
Adrenaline	-0.57	0.018*
Tranexamic acid	-0.31	0.025*
Pressure gauze	0.72	0.035*

## Discussion

Effective management of bleeding following dental extraction is crucial to ensure patient comfort, prevent complications, and facilitate wound healing. In this RCT, we compared the hemostatic efficacy of four local agents, Botroclot (hemocoagulase), chitosan, adrenaline, and tranexamic acid, with that of conventional pressure gauze dipped in normal saline to control post-extraction bleeding.

The observation time, defined as the duration for which bleeding was monitored after application of the agent, showed statistically significant differences among the groups (p = 0.001), with the pressure gauze group demonstrating the longest observation time (1.80 ± 0.41 minutes). This finding supports the hypothesis that passive mechanical pressure with saline-soaked gauze is the least effective modality for achieving rapid hemostasis [15]. Levi et al. [15] suggested extended pressure using a shaver blade to achieve hemostasis in three seconds.

Botroclot, a hemocoagulase derived from snake venom, has the shortest observation time and is the most effective hemostatic agent. The mechanism of Botroclot involves activation of coagulation pathways and enhancement of fibrin formation, which aids in clot stabilization [5]. Hemocoagulase functions by inducing the cleavage of fibrinogen into fibrin monomers, ultimately leading to the synthesis of fibrin polymers [16]. The characteristics of BTX are distinctly different from those of thrombin, as are the resulting clots. BTX exhibits systemic and localized activity even in the absence of critical clotting factors. BTX has been demonstrated to facilitate wound healing in dental surgical procedures, primarily by enhancing collagen accumulation while simultaneously diminishing inflammation and the risk of infection. In instances of primary intention healing, BTX contributes to the effective closure of wound edges and mitigates scar tissue [6].

Tranexamic acid, an antifibrinolytic agent, also demonstrated good hemostatic control, comparable to that of Botroclot, with a relatively short observation time. By inhibiting the conversion of plasminogen to plasmin, tranexamic acid stabilizes fibrin clots, thereby reducing bleeding [10,17]. This property is particularly useful in patients at risk of prolonged bleeding, such as those on anticoagulant therapy [17]. Although such patients were excluded from our study, the favorable results obtained in healthy individuals suggest the potential utility of tranexamic acid in more complex patient populations.

The results of the present study further indicate that chitosan also demonstrated superior hemostatic action, which can be attributed to its unique cationic properties that enable it to interact with negatively charged red blood cell membranes, promoting rapid clot formation [7]. Furthermore, chitosan has inherent antibacterial and wound-healing properties, which may contribute to improved postoperative outcomes. It is widely acknowledged that the cationic characteristics of the -NH3+ moieties present in chitosan enhance the adhesion and aggregation of erythrocytes and platelets [18]. Contemporary investigations have revealed that chitosan can stimulate platelet activation through Toll-like receptor 2, thereby promoting hemostatic processes in patients undergoing oral antiplatelet therapy [19]. Huang et al. [20] reported a better hemostatic effect with protonated chitosan than with commercially available chitosan.

The control group, which relied on pressure gauze soaked in normal saline, consistently underperformed across all parameters. It demonstrated the longest observation time, and its comparison with all other test groups (except for some non-significant comparisons with adrenaline) yielded statistically significant differences. This reinforces the notion that, while pressure gauze may eventually control bleeding via mechanical tamponade and natural coagulation, it is neither efficient nor reliable compared to pharmacologically active agents [15].

Correlation analysis revealed significant associations between BT and observation time in the adrenaline, tranexamic acid, and pressure gauze groups, but not in the Botroclot and chitosan groups. This suggests that the effectiveness of adrenaline and tranexamic acid is more closely related to the duration of their application or action, whereas Botroclot and chitosan may exert their effects more promptly and independently. In particular, the lack of a significant correlation in the chitosan group supports its rapid-action hemostatic property, possibly making it an ideal choice in clinical settings requiring quick intervention [21]. Mahardawi et al. [21] conducted a systematic review and meta-analysis to study the effects of different hemostatic agents following dental extractions. The authors included 22 studies and concluded that although tranexamic acid was a good hemostatic agent, chitosan outperformed all other agents.

No adverse events were reported in terms of safety, and no cases required interim analysis, confirming the clinical safety of all tested agents within the study’s sample population. This aligns with the existing literature, which has consistently reported minimal complications associated with the topical application of chitosan, Botroclot, and tranexamic acid [22].

From a clinical standpoint, this study supports the use of chitosan, tranexamic acid, and Botroclot as preferable alternatives to conventional pressure gauze, particularly when rapid hemostasis is desired. Despite its common use, adrenaline may require re-evaluation given its potential systemic effects [23].

The strength of the current study lies in its rigorous design, including randomized group allocation, allocation concealment, blinding of outcome assessors, and use of standardized procedures by a single operator. However, this study had certain limitations. Although statistically adequate, the sample size was limited to 60 patients, restricting subgroup analysis (such as sex-based or age-specific differences). In addition, the study was conducted only in systemically healthy individuals, which limits its generalizability to patients with bleeding disorders or those receiving anticoagulant therapy. Future studies with larger sample sizes and more diverse patient populations are required to validate these findings.

## Conclusions

Within the limitations of this RCT, Botroclot emerged as the most effective local hemostatic agent for achieving rapid hemostasis following minor oral surgical procedures, with a significantly reduced observation time compared to adrenaline and conventional pressure gauze. Tranexamic acid and chitosan also demonstrated superior hemostatic performance compared to the control group, although the differences were not statistically significant. Pressure gauze showed the longest observation time to achieve hemostasis, confirming its relatively lower efficacy. No postoperative bleeding or adverse effects were observed in any group, indicating the safety of all the tested agents. These findings suggested that Botroclot, tranexamic acid, and chitosan can be recommended as efficient and reliable alternatives to traditional hemostatic methods in routine clinical oral surgery.
